# Effects of Arsenic, Iron and Fertilizers in Soil on Rice in Cambodia

**DOI:** 10.5696/2156-9614-8.19.180910

**Published:** 2018-09-10

**Authors:** Tom Murphy, Kongkea Phan, Emmanuel Yumvihoze, Kim Irvine, Ken Wilson, David Lean, Alexander Poulain, Brian Laird, Laurie Hing Man Chan

**Affiliations:** 1 International University, Phnom Penh, Cambodia; 2 University of Ottawa, Canada; 3 Nanyang Technological University, Singapore; 4 Texas State University, San Marcos, Texas, USA; 5 Lean Environmental, Apsley, Ontario, Canada; 6 University of Waterloo, Canada

**Keywords:** arsenic, remediation, bioaccumulation, irrigation, dimethylarsinic acid, rice

## Abstract

**Background.:**

In parts of Cambodia, irrigation with groundwater results in arsenic accumulation in soils and rice, leading to health concerns associated with rice consumption. A high concentration of iron in groundwater can precipitate arsenic and reduce its bioavailability, however high concentrations of arsenic and iron can also reduce rice production. Furthermore, concerns have been raised about chemical contamination from inorganic fertilizers used to grow rice. The relationship between soil geochemistry and arsenic concentrations in rice is not yet fully understood.

**Objectives.:**

The primary objective of this project was to investigate the relationship between arsenic concentrations in irrigation water, soil and rice collected from different sites in Cambodia. A secondary objective was to explore arsenic and phosphorus levels in fertilizer samples obtained from the study area in Cambodia.

**Methods.:**

The present study collected 61 well water samples, 105 rice samples, 70 soil samples, 11 inorganic fertilizer samples and conducted interviews with 44 families along the Mekong River in Cambodia. Analyses for metals, total arsenic, and arsenic species in the water and rice were conducted in Canada by inductively coupled plasma mass spectrometry. Analyses for metals, total arsenic and phosphorus in soils and inorganic fertilizers were conducted in Cambodia and Singapore by X-ray fluorescence.

**Results.:**

The concentration of arsenic in rice paddy soils was highly variable and as much as 20 times higher near the irrigation wells than in more distal areas of the paddy. Two farmers in Preak Russey had integrated soil samples with arsenic levels above the concentration associated with toxicity to rice in Taiwan (40 mg/kg) and above the Dutch concentration requiring intervention or remediation (55 mg/kg). The highest total arsenic measured in soil was 95 mg/kg. In Preak Russey, the loading of arsenic from irrigation water was 3710 times greater than the loading of arsenic from inorganic fertilizers. Half of the commercial inorganic fertilizers had less than 50% of the labelled content of phosphorus.

**Conclusions.:**

Emphasis should be placed on improving the management of irrigation water, not on inactivation of arsenic in soil. The high levels of iron in groundwater mitigate arsenic toxicity, but the accumulation of iron could later result in lower rice productivity. Irrigation of rice with groundwater is not likely sustainable. To improve rice productivity, the content of phosphorus in local inorganic fertilizers must be improved to world standards. X-ray fluorescence analysis can quickly identify poor quality fertilizers.

**Informed Consent.:**

Obtained

**Competing Interests.:**

The authors declare no competing financial interests

## Introduction

Arsenic bioaccumulation in rice is a serious threat to agricultural production and public health.^1^,^2^ The primary source of arsenic in Cambodia, Bangladesh, Vietnam and other countries in South and Southeast Asia is oxidation of arsenopyrite that naturally occurs in the Himalayan Mountains. The importance of local metamorphic rocks with arsenopyrite in Cambodia is not fully documented and likely will become more important as more of these rock outcroppings are exploited for construction materials.

The arsenic in soils is relatively stable and dissolution of arsenic into groundwater is largely mediated by introduction of iron or fresh organic matter to groundwater.^3^ In the areas of greatest arsenic contamination in Cambodian rice, arsenic primarily reflects use of groundwater for irrigation.^4^ It is generally believed that it takes years of irrigation with groundwater for soil to be contaminated with arsenic before rice becomes badly contaminated with arsenic.^5^ Groundwater is initially anoxic and highly enriched with both arsenic and iron. When the irrigated water is oxygenated, the high concentrations of iron in irrigation water initially precipitates arsenic. In fields, the solubility of the arsenic is increased when the oxygen concentration decreases as the fields are flooded in the growing period. Growing rice in oxygenated soil markedly reduces arsenic bioaccumulation.^6^ There are systems of rice cultivation such as the system of rice intensification (SRI) that use less water, produce oxic growing conditions and can significantly decrease arsenic bioaccumulation.^7^ However, it has been reported that Cambodian farmers are hesitant to release water that they may later need and also prefer not to use the SRI method due to increased labor requirements.^8^

Globally, there are places where agricultural chemicals have resulted in arsenic contamination. Dimethylarsinic acid (DMA) was used extensively as a pesticide on cotton, which was an alternative crop with rice in the southern United States.^9^ Over 30,000 tons of DMA were applied to about 40,000 km^2^ of American cotton/rice fields; however, 20–30 years after DMA was banned in cotton fields, arsenic still persists and is a concern.^9^ It is noteworthy that in the Vietnam War, 74,000 tons of DMA were dropped onto 2000 km^2^ of Vietnam rice paddies.^10^ In addition, as phosphate sources are becoming depleted, there is growing concern that phosphate rock contaminated with arsenic, cadmium or other toxic metals will be used.^11^,^12^ Cambodian farmers believe that nitrogen-phosphorus-potassium (NPK) fertilizers in Cambodia are intentionally diluted with inert or contaminated materials and placed in bags so as to appear to be reputable products. There is some evidence to support this belief.^13^ Jayasumana et al. reported that arsenic in inorganic fertilizers used to grow rice were a possible cause of kidney cancer in Sri Lanka.^14^ The primary objective of this project was to investigate the relationship between arsenic concentrations in irrigation water, soil and rice collected from different sites in Cambodia. A secondary objective was to explore arsenic and phosphorus levels in fertilizer samples obtained from the study area in Cambodia.

**Abbreviations**
*IRDC*: International Development Research Centre
*LOD*: Limit of detection
*NPK*: Nitrogen phosphorus potassium
*SRI*: System for rice intensification
*XRF*: X-ray fluorescence

## Methods

**Figure 1:**
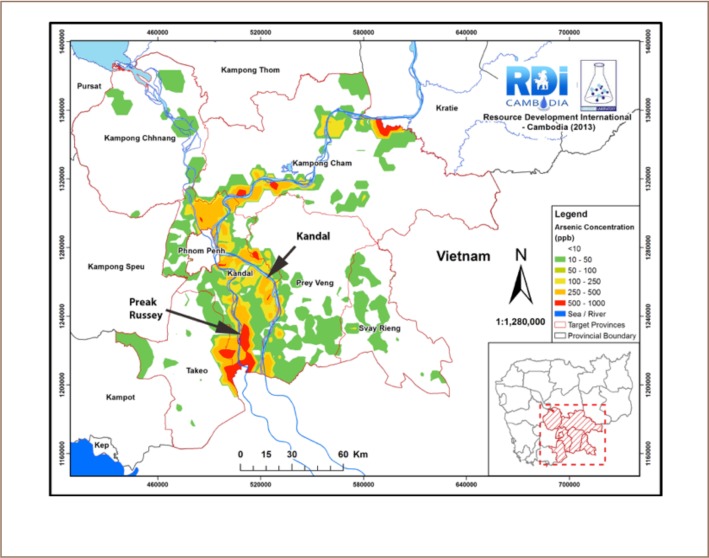
Primary sampling sites

The present study evaluated a site of high arsenic contamination, Preak Russey near the Bassac River and a less contaminated area of Kandal Province near the main branch of the Mekong River (*Figure 1*). The less contaminated site is referred to as the Kandal site in the present analysis. Details of the sites, sample collection and processing can be found in our International Development Research Centre, Canada (IDRC) report.^4^,^15^ Efforts were made to collect rice at every site where soil or water samples were collected and interviews were conducted in the present study. In total, 102 rice samples in husks (paddy rice) were collected.

### Soil analysis

Twenty-four soil samples were collected from the Kandal site. Fifty soil samples were collected from Preak Russey. A small Cambodian digging tool was used to extract soil plugs 10 cm deep and 4 cm wide. Typically for bulk integrated samples, each field had samples collected at three sites (>30 m apart), with one near the irrigation well and at each sampling site, triplicate samples (2 m apart) were collected to be combined, i.e. 9 scoops were combined in each field. For sites Preak Russey-2 and Preak Russey-9, 4 sets of samples were collected for a limited spatial analysis. Soil samples were air dried and then ground with a mortar and pestle. A set of 10 soil samples were freeze-dried to confirm that the soils were dry. Samples were sieved through a 100-μm mesh. Soil samples were typically greater than 300 g.

### Analysis of soils and fertilizers

**Figure 2:**
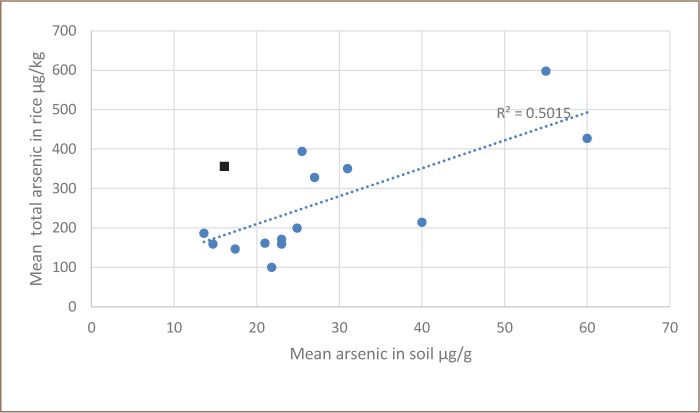
Total arsenic in rice versus total arsenic in irrigation water The square represents Preak Russey-1.

Arsenic in soil was measured with a Niton XL3tGOLDD handheld analyzer in Cambodia. The certified reference materials supplied by Thermo Fisher Scientific were used in each set of samples. The counting time was usually one or two minutes, but at times was increased up to 10 minutes. All samples were processed using the sample cup method recommended by Thermo Fisher Scientific with Mylar film (Figure 2, Appendix 1 of the IRDC report).^15^,^16^ The measured mean and standard deviation of X-ray fluorescence (XRF) analysis of four certified reference materials (CRMs) were 9±12% of the certified values (*Table 1*). The means were within 3% of the certified values for the two CRMs closest in concentration to the samples. These CRMs were all soil samples. Although some studies have reported that CRMs with different matrices worked well with XRF analysis, the present study found that CRMs with an organic matrix did not produce satisfactory results. The XRF sensor had already been optimized for arsenic analysis by the supplier which enhanced accuracy of arsenic analyses. Statistical analyses used Excel and VassarStats.^17^

**Table 1:**
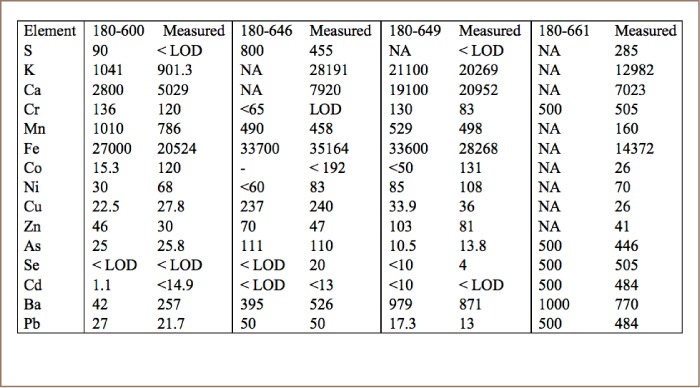
Analysis of Certified Reference Materials (180–600, 180–646, 180–649, 180–661) in Soil Mode (ppm), Thermo 2017

## Results

Irrigation of rice with arsenic-rich groundwater has occurred for 1 to 34 years in the study area (Table 5, Appendix 2 of the IRDC report).^15^ In spite of the variation in years of irrigation, there was a significant correlation between the total arsenic content of the rice grain and the total arsenic in soil (p>0.01, r^2^=0.502) (*Figure 2*). An earlier study in Preak Russey showed a slightly stronger relationship (r^2^ = 0.682) between the total arsenic content of rice grain and arsenic of soil.^18^ There was one outlier sample from site Preak Russey-1. If the Preak Russey-1 result was removed, the regression coefficient of determination increased to 0.784. The rice from this Preak Russey-1 field had much greater total arsenic concentration relative to the soil content of arsenic of other farms. The rice at Preak Russey-1 was yellowish, indicating chlorosis. Arsenic toxicity can produce scorching of leaves.^19^ But the arsenic level in the soil of Preak Russey-1 was one of the lowest in Preak Russey, and the reported rice production was not reduced (Table 5, Appendix 2 of the IRDC report).^15^ If the chlorosis at Preak Russey-1 did reflect arsenic toxicity, there was likely another unknown variable enhancing the arsenic toxicity. There was nothing obviously unusual noted about the farm management, but we were unable to evaluate parasites, phosphorus or levels of other soil nutrients such as zinc, sulfur, silicon, potassium, or nitrogen on most farms. Low soil phosphorus content is a potential mechanism by which arsenic in rice may become elevated at a particular site. However, soil phosphorus analyses (as described below) suggest an alternate mechanism behind the higher arsenic bioaccumulation rates observed in Preak Russey-1.

**Figure 3:**
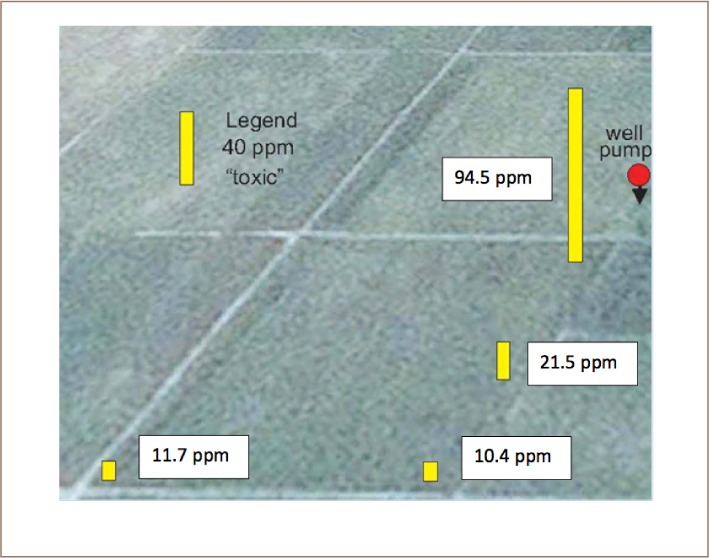
Spatial variability in soil arsenic relative to the well site Preak Russey-2. Field is 53 m wide

**Figure 4:**
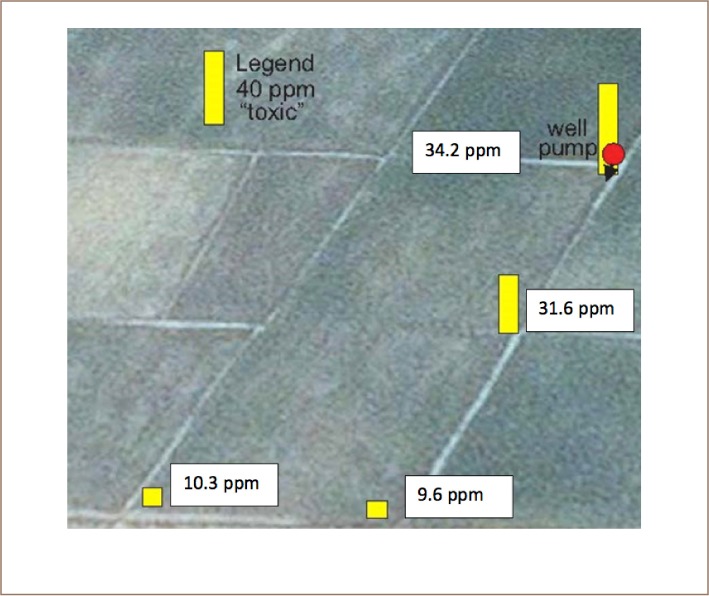
Spatial variability in soil arsenic relative to the well site Preak Russey-9

**Figure 5:**
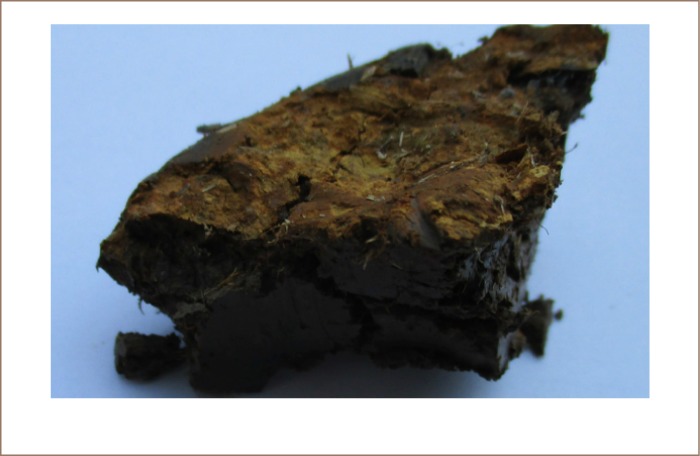
Surface of Preak Russey-10 soil stained red and yellow from iron precipitation

**Figure 6:**
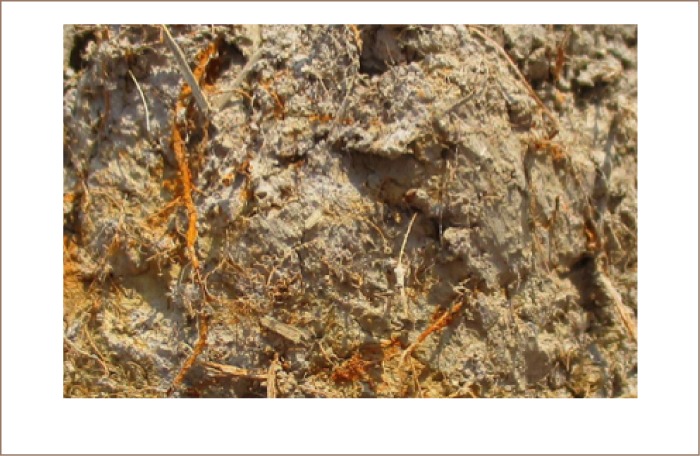
Intense precipitation of iron on rice roots. The highly reactive iron will control most nutrient and toxin availability

The worst soil found in Preak Russey contained 95 mg/kg of arsenic, almost twice that of the intervention value/indicative level for serious contamination in Holland.^20^ The concentration of arsenic in the rice paddy soils was highly variable and much higher near the irrigation wells (*Figure 3 and 4*). Precipitation near the irrigation wells of Preak Russey is driven by the high concentration of iron in groundwater (9565±6635 μg/L, n=20) and once iron is exposed to the atmosphere, arsenic readily precipitates with iron. Figure 5 shows that the soil surface was reddish or yellow in places due to iron precipitation. The red color likely reflects hematite, a ferric oxide. The yellow color is likely limonite, a hydrated ferric oxide. Perhaps even more illustrative is the intense precipitation of iron on the roots of rice (*Figure 6*). This iron precipitation will strongly influence the biogeochemistry of arsenic, phosphorus, zinc and other nutrients and toxins. Although iron suppresses arsenic toxicity, such soils that are stained by iron from groundwater irrigation are impacted in negative ways as well. CEDAC, a Cambodian NGO that trains and manages about 160,000 farming families believes that such iron stained soils, even outside of the arsenic zone are usually unproductive.^21^

The mean concentration of total arsenic in the integrated soil samples from Kandal was 13.1±3.5 mg/kg (n=8), as shown in Tables 5 and 6, Appendix 1 of the IRDC report.^15^ By comparison, the mean concentration of total arsenic in integrated soil samples from Preak Russey was 24±16.9 mg/kg (n=19), as shown in Tables 7 and 8 Appendix 1 of the IRDC report.^15^ The most important observation is that two farmers in Preak Russey (Preak Russey-13, Preak Russey-10) had integrated soil samples with arsenic above the concentration associated with toxicity to rice in Taiwan (40 mg/kg).^22^ They are also above the Dutch guideline concentration, in effect since 2000, requiring consideration of intervention or remediation (55 mg/kg).^20^

### Phytotoxicity

There was enhanced bioaccumulation of arsenic into rice in Preak Russey relative to the much less contaminated farms in the Kandal sites.^4^ The average total arsenic content of rice and irrigation wells in Preak Russey was 315±150 μg/kg and 959±351 μg/L vs. 158±33 μg/kg and 65±51 μg/L, respectively, in Kandal (Table 2, Appendix 2 of the IRDC report).^15^ By comparison, there was higher rice productivity in Preak Russey (5.6±1.6 t/ha, n=12) compared to the Kandal sites (4.0±1.6 t/ha, n=8). There was significantly enhanced bioaccumulation of arsenic, but no reduction in rice production from irrigation water (Mann-Whitney U test, α=0.05). The Kandal and Preak Russey sites are similar clay flood plains, but variability in farm management and natural detoxification reactions made Kandal a weaker control with respect to phytotoxicity.

**Table 2:**
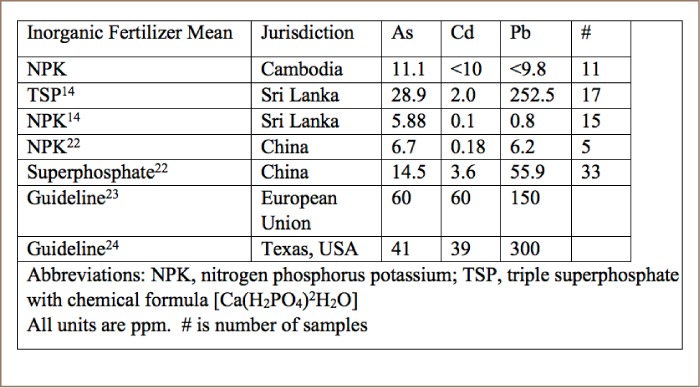
Comparison of Metals in Inorganic Fertilizers and Guidelines

Regardless, there remains anecdotal evidence suggesting that current arsenic loadings may lead to phytotoxicity. The farmer at site Preak Russey-15 had been growing rice for 20 years on two fields, one with surface water only and one using groundwater in the dry season. With the same farming practices, he observed a 29% greater productivity in the field irrigated with surface water. The suppressed productivity in the field irrigated with groundwater may represent arsenic toxicity, but iron toxicity is also possible. Both farmers at sites Preak Russey-4 and Preak Russey-17 claimed that when they were unable to dilute the groundwater with surface water, their rice productivity decreased and irrigation of cucumbers with 100% groundwater resulted in no crop production.

### Iron loadings to soil

Especially in Preak Russey, the loading of iron to the soils was quite large and likely restricted most arsenic phytotoxicity to near the wells. Using the average irrigation volume of 11,600 m^3^/ha, the loading of iron can be calculated as 111 kg/ha per year. Using the formula of FeO(OH)-6H_2_O for limonite, the loading of this iron mineral is estimated to be 266 kg/ha per year. Limonite is a hydrated hematite that is often called “bog iron”. Its yellow color is commonly seen in soils that are irrigated with groundwater.

### Phosphorus limitations, other metals, and potential effects on rice

**Figure 7:**
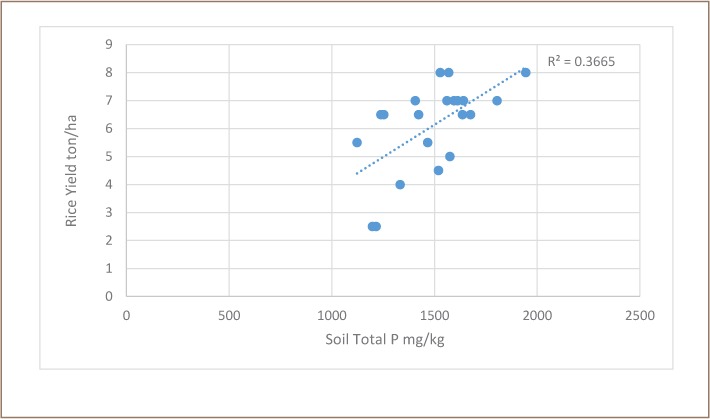
Effect of phosphorus in soil on rice production

The effect of soil phosphorus content on reported rice production was significant (t-test for slope of regression was greater than 0 at α=0.05, Figure 7), but not strong (r^2^ =0.3665). The concentration of phosphorus in the soils of site Preak Russey-1 was the 3rd highest measured; this site had higher than average rice productivity, and there is no support for the hypothesis discussed earlier that phosphorus limitation might have caused the enhanced arsenic bioaccumulation relative to the soil content of arsenic in Preak Russey-1. The three-fold variation in rice production in Figure 7 is much greater than the modest and only occasionally apparent suppression of rice production by irrigation with arsenic in this study. The apparent arsenic anomaly suggests that most arsenic from irrigation precipitates near the wells and most of the rice productivity is not strongly affected by the arsenic in the wells.

**Figure 8:**
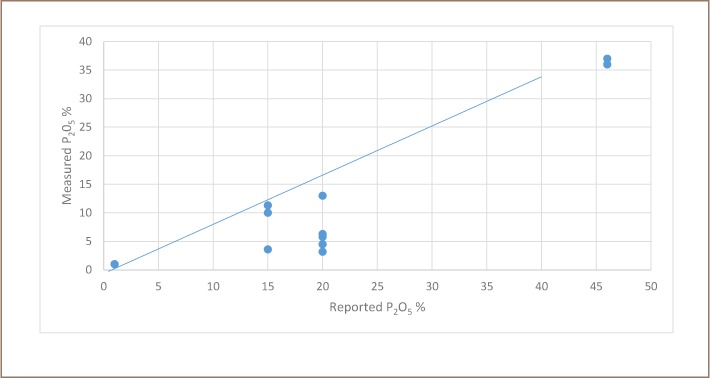
Comparison of measured and claimed phosphorus content of fertilizers. The drawn line connects samples with a response predicted by the labelled content and is not a statistical relationship including the deviant samples.

It has been reported that NPK blends originating from Vietnam and sold in Cambodia were deficient in nutrient content.^13^ The research site was dominated by Vietnamese NPK suppliers and our more recent analysis confirmed the findings of the World Bank study suggesting that this low-quality fertilizer problem persists.^13^ Our study of 11 chemical fertilizers indicated that half of the fertilizers had <50% less phosphorus than labelled (*Figure 8*). As with the World Bank study, the diammonium phosphate (DAP) samples had somewhat lower phosphate content than claimed (*Figure 8*).^13^

Fortunately, the concentration of toxic metals in NPK fertilizers we analyzed was only a minor problem (*Table 2*). One of the DAP samples from China had 72.5 ppm of arsenic which fails two guidelines (*Table 3*). The other 10 samples passed European Union (EU) and State of Texas guidelines.^23^,^24^ The XRF analysis indicated that fertilizer cadmium levels were lower than EU or Texas guidelines (*Table 2*).^23^,^24^ The Cambodian NPK fertilizers had less arsenic, cadmium or lead than Chinese DAP.^25^ Based on the sampling results for Preak Russey and interviews, the present study calculated the loading of arsenic from irrigation water to be 3710 times greater than the loading of arsenic from inorganic fertilizers (*Table 4*).

**Table 3:**
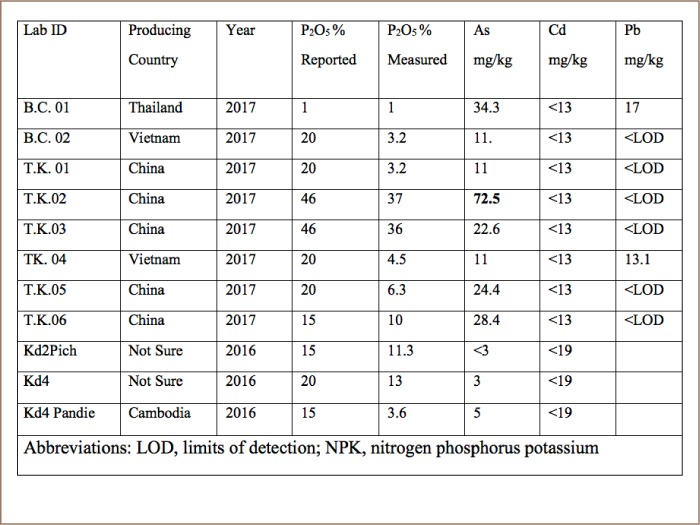
Metal Contamination in NPK Fertilizers Collected in Cambodia

**Table 4:**
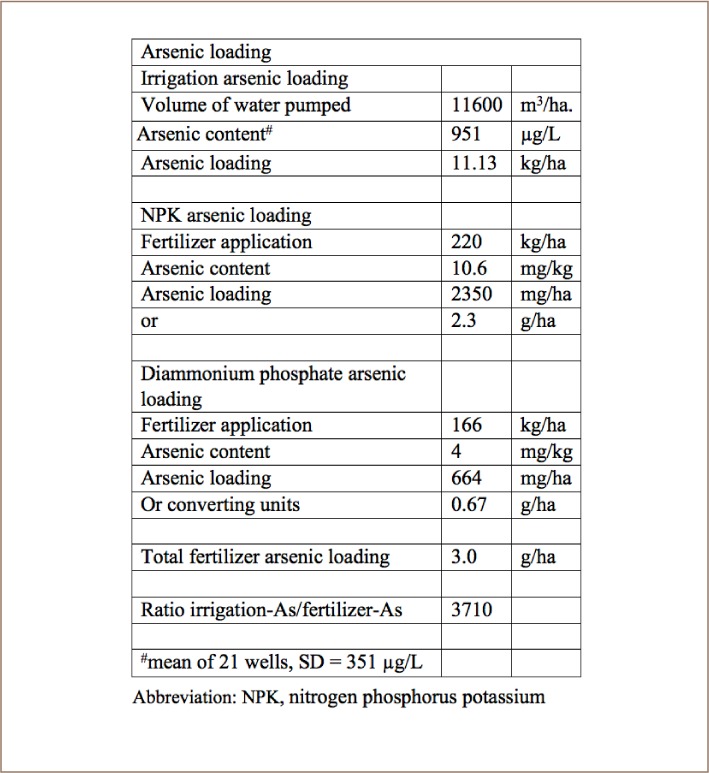
Arsenic Loadings to a “Typical” Field from Preak Russey^*^

## Discussion

Although arsenic in groundwater and soil are the primary variables influencing arsenic bioaccumulation in rice, other variables influence this relationship and the correlation between arsenic in rice and arsenic in soil is not always strong. Rahman found that the arsenic content of rice grain was not significantly correlated to the soil arsenic concentration.^26^ Panaullah et al. reported a good relationship between soil arsenic and rice grain in the second year, but not the first year of their project.^27^ The differences between studies are not yet clear. Some variables influencing arsenic bioaccumulation vary from year to year. In our study, the intensity of monsoons, availability of surface water, and quality of inorganic fertilizers varied considerably and could all affect arsenic contamination.

The Dutch soil remediation intervention values “indicate when the functional properties of the soil for humans, plant and animal life are seriously impaired or threatened”.^20^ In 2015, Holland proposed a new guideline for maximum permissible addition of arsenic of 0.0012 mg/kg.^28^ If this increment was based on the loading of arsenic, it would not permit use of groundwater irrigation at Preak Russey, Cambodia.

The net incremental addition of arsenic to soils is not simple to predict from loading measurements. Loss rates from erosion or volatilization are difficult to measure quantitatively. In Bangladesh, 24–48% of the arsenic annually added to the study field from irrigation was removed from the soil during the monsoon season.^29^ The data from the present study is less extensive, but at least qualitatively it seems that less arsenic was retained in Preak Russey soils. Our soil cores were 10 cm deep. In May 2016, the soils were drained and oxic, which resulted in the red color of the roots from iron oxide being strongly contrasted to the grey soils (*Figure 6*). At least 90% of the roots appeared to be less than 10 cm deep. Our depth of sample cores was shorter than ideal, but allow for simple estimates. Duxbury and Roberts showed that in Bangladesh, about 30% of the arsenic would be deeper than 10 cm.^7^,^30^ Our samples were 10 cm deep, and to be conservative, we doubled the amount of arsenic that was measured for a preliminary calculation of arsenic retention. This calculation suggests total arsenic export from the fields since the start of irrigation to be 96.5% for Preak Russey-2 and 95.5% for Preak Russey-9 fields. We also processed integrated soil sample cores from five fields and calculated that 96.5±4.0% of the arsenic added by irrigation was lost since groundwater irrigation began (3–13 years), Appendix 2 of the IRDC report.^15^ If we were to go to the extreme of assuming that all soils were as contaminated as the worst soils measured near the wells, the retention of arsenic in the fields would still be less than 10% of what was loaded. Future work should collect more soil cores (~30/ha), evaluate the concentration of arsenic in deeper samples and use the kriging method for statistical analysis.

Spatial analysis is also needed for assessment of phytotoxicity from well water. Phytotoxicity would likely be better assessed by comparing rice productivity with associated arsenic in soil and water samples at varying distances from the irrigation wells. The spatial variability of arsenic accumulation in soil is not commonly measured, but it is very important in evaluating the effect of irrigation water.^5^,^29^,^30^ Abedin et al. observed a 23% reduction in production of potted rice when irrigated with water having arsenic at a concentration of 1 mg/L, roughly the same arsenic concentration in irrigation water as used by Preak Russey-15.^31^ Azad et al. found a 53% reduction in rice production from an arsenic solution of 1 mg/L.^32^ However, these controlled experiments in pots do not necessarily represent the responses of field conditions where most arsenic precipitates near the wells.

### Iron remediation options

Studies have suggested that addition of iron to paddy fields could overcome arsenic toxicity.^33–35^ In Cambodia it would be possible to grind an iron-rich laterite and use it as an amendment to paddy soils. Laterite deposits in this region have an average iron content of 22.5%, so with the appropriate correction for the stoichiometry of the iron mineral for the equivalent iron loading as comes from the groundwater irrigation, a farmer would have to add 489 kg/ha per year of laterite.^36^ Moreover, the iron in the laterite would not be nearly as reactive as the iron in the groundwater, and for the same effect, a much larger laterite dosage would be required. The potential “effective” dose of laterite to inactivate arsenic could easily be >1000 kg/ha/yr. The effective dose of three iron-rich materials (25%, 56% and 99% iron) used to block arsenic assimilation by rice in Japan was 5000 kg/ha.^33^ Excavation, grinding and shipping this amount of laterite from a deposit in Mondulkurri (a mountainous area of Cambodia) is likely prohibitively expensive for rice farmers.

Iron additions to soils can reduce arsenic toxicity, but the iron will mostly persist in soils. This is the natural geochemistry producing bog iron deposits. There are strong reasons to be concerned about the long-term toxicity of high iron applications, including current irrigation with groundwater and suggested remedial options with iron. High levels of iron in soil both block nutrient assimilation and produce oxidative stress in plants.^37^,^38^ Iron toxicity has been identified in rice paddies in Cambodia, India, Indonesia, Malaysia and the Philippines.^20^,^39^,^40^ Rice fields are prone to iron toxicity because of the anoxia resulting from long periods of flooding that dissolves iron. Relative to the extensive publications on arsenic toxicity, iron toxicity does not get much attention. However, iron toxicity mainly affects rice productivity and iron is rarely a toxic concern to humans. Even outside of the arsenic-rich zone, the Cambodian Center for Study and Development in Agriculture (CEDAC) discourages the use of groundwater for irrigation because it forms yellow soils of low productivity.^21^ Any irrigation technique that results in extensive periods of standing water and anoxia will produce iron toxicity.^41^ The SRI approach that CEDAC encourages would decrease iron toxicity significantly, because this technique does not flood soils for extensive periods which results in low oxygen and high iron solubility.^20^ However, consultations with ~300 farmers could not find anyone in the arsenic-rich zone using SRI. Two sets of farmers in the arsenic-rich zone who had been trained and used the SRI method complained that it required more weeding. Storage of surface water for irrigation which is also recommended by CEDAC seems a better option.

In India, iron toxicity is treated by addition of lime or potassium.^40^ Usually, potassium is not considered as important as phosphorus and nitrogen in rice production, but it is more likely to be important in iron-rich soils, especially for farmers using only diammonium phosphate. The Rice Institute states that there is no practical field management option to treat iron toxicity.”^41^ Iron toxicity is best avoided.

Phosphorus amendments are also often discussed or proposed as a method to overcome arsenic toxicity and block arsenic bioaccumulation (Appendix 3 of the IRDC report).^15^,^34^ In Cambodia, it is premature to consider using phosphorus to remediate arsenic contamination. Moreover, the possibility of arsenic treatment with good quality phosphorus fertilizers is uncertain (Appendix 3 of the IRDC report).^15^

The quality and price of inorganic fertilizers must first be improved. When possible, organic fertilizers should be encouraged. Phosphorus is important for basic rice production. Phosphorus is the most limiting nutrient for rice production in clay soils of Southeast Asia.^42^,^43^ As well as the poor quality of many chemical fertilizers, fertilization management affects Cambodian farmers in other ways. Cambodian farmers pay 50% more for inorganic fertilizers than international rates.^13^ Moreover, farmers we interviewed complained about the high interest rates required to buy fertilizers on loan and repay at rice harvest.

Conversely, one common complaint about fertilizers appears unfounded. It appears that fertilizers are a relatively minor source of arsenic contamination in Cambodia. Further analysis should be conducted on NPK fertilizers to confirm the generalizability of these results.

### Phosphorus analysis

Our extrapolation of the present results and those of the World Bank study for this region of Cambodia which is dominated by suppliers of poor inorganic fertilizers predicts an economic loss of millions of dollars per year in rice productivity.^13^ The financial loss and associated impairment of food supplies could be multiplied considerably if similar fertilizers are also used elsewhere in Southeast Asia. Anecdotal information suggests there is less phosphorus than labeled in inorganic fertilizers in Indonesia as well. The XRF results of the present study have been confirmed by a second laboratory in Singapore and we are examining the persistence of a serious problem identified in a much larger study by the World Bank.^13^ The validation of results is always important, but XRF analysis appears to be a convenient technique to monitor NPK quality. In minutes, XRF analysis can identify a lack of phosphorus in inorganic chemical fertilizers with no wet chemistry required. The sample matrix can interfere more with XRF analysis of phosphorus than it does for arsenic or other heavy metals. The linearity of the responses for phosphorus as shown in Figures 3 and 4, Appendix 3 of the IRDC report was excellent, but confirmation of results is essential.^15^ Future work should include confirmation of the XRF analysis with extractions and spectrophotometric analysis and geographic expansion of the data set.

## Conclusions

The concentration of arsenic in the rice paddy soils was as much as 20 times higher near the irrigation wells. The most contaminated soils contained 95 mg/kg of arsenic, which is more than twice the concentration associated with toxicity to rice in Taiwan (40 mg/kg) and almost twice the Dutch concentration requiring planning for intervention or remediation (55 mg/kg). The rice has more arsenic when the concentration of arsenic in soil is high, but still passes Codex guidelines. In Preak Russey, the loading of arsenic from irrigation water was 3710 times greater than the loading of arsenic from inorganic fertilizers. Half of the commercial inorganic fertilizers analyzed in this study had less than 50% of the labelled content of phosphorus. Emphasis should be placed on improving the management of irrigation water, not the inactivation of arsenic in soil.
